# The anti-hyperglycemic efficacy of a lipid-lowering drug Daming capsule and the underlying signaling mechanisms in a rat model of diabetes mellitus

**DOI:** 10.1038/srep34284

**Published:** 2016-10-10

**Authors:** Yong Zhang, Xiaoguang Li, Jiamin Li, Qingwei Zhang, Xiaohui Chen, Xin Liu, Yue Zhang, Haiying Zhang, Huan Yang, Yingying Hu, Xianxian Wu, Xin Li, Jiaming Ju, Baofeng Yang

**Affiliations:** 1 Department of Pharmacology (State-Province Key Laboratories of Biomedicine-Pharmaceutics of China, Key Laboratory of Cardiovascular Medicine Research, Ministry of Education), College of Pharmacy, Harbin Medical University, Harbin, 150081, China; 2 Institute of Cardiovascular Research, Harbin Medical University, Harbin 150081, China; 3 Institute of Metabolic Disease, Heilongjiang Academy of Medical Science, Harbin 150086, China; 4 Department of Pharmacology and Therapeutics, Melbourne School of Biomedical Sciences, Faculty of Medicine, Dentistry and Health Sciences, University of Melbourne, Melbourne, Australia

## Abstract

Diabetes mellitus (DM) is a metabolic disorder manifested by hyperglycemia. Daming Capsule (DMC), a combination of traditional Chinese herbs, is used clinically as a lipid-lowering drug. This study was designed to evaluate if DMC possesses an anti-hyperglycemic effect and to elucidate the underlying mechanisms. Compared to diabetic rats, the rats received DMC (200 mg/kg/d) had significantly lower blood lipid and glucose levels. DMC markedly restored the decreased secretion of GLP-1 and GIP as well as the coding gene GCG and GIP in ileum. Moreover, DMC normalized depressed GCG and GIP transcription by significantly enhancing the GSK-3β/β-catenin signaling pathway and expression of TCF7L2, a transactivator of GCG and GIP in diabetic rats. DMC possesses an anti-hyperglycemic property characterized by preservation/stimulation of GLP-1 and GIP secretion in DM rats. Here, we proposed DMC → GSK-3β/β-catenin↑ → TCF7L2↑ → GLP-1, GIP secretion↑ → blood glucose↓ as a regulatory pathway of blood glucose homeostasis. Our findings suggest DMC as a promising therapeutic drug in the clinical treatment of diabetes.

Diabetes mellitus (DM) is a chronic metabolic disorder afflicting a large population of human beings. The first sign of DM is hyperglycemia along with progressive decreases in insulin secretion due to dysfunction of pancreas or loss of response of cells to insulin. Epidemiology of DM exhibits a stable uptrend development worldwide, especially in the developing countries. The number of diabetics has reached up to 200 million globally and it is anticipated to double by the year 2025 according to WHO reports^1^,^2^,^3^,^4^. DM has become the third biggest threat to human health after cardiovascular disease and cancer, and is projected to become one of the world’s main killers in the next 25 years^5^. However, there is no optimal form of definitive therapy for diabetes at present and its treatments may cause many complications. There is therefore an urgent need to develop complementary and alternative medicine with minimal side effects.

Traditional Chinese medicine (TCM) has demonstrated superior efficacy (stable curative effects and low toxicity) in treating DM over the anti-diabetic drugs currently in clinical use^6^. The growing popularity of TCM for DM is fueled by increasing scientific interest in herbal medicines which are generally used as a combination of disparate herbs based on traditional oriental medical theory^7^. Daming Capsule (DMC), a mixture of traditional Chinese herbs, is formulated in accordance with the TCM theory comprising several ingredients mainly including Rheum palmatum L. (Solanaceae), Cassia obtusifolia L. (pulse family), Salvia miltiorrhiza Bunge. (Labiatae), and Panax ginseng C. A. Meyer (Acanthopanax gracilistylus) (Supplemental Table 1)^5^. Proportional mix of these herbs can synergize the desirable medicinal properties such as lipid-lowing effect and minimize side effects and toxicity. Our previous study demonstrated promising efficacy and safety profile of DMC in patients with hypercholesterolemia^5^,^8^,^9^,^10^,^11^. We have also identified the protective effects of DMC against impairment of baroreflex and heart function, and restoration of endothelial dysfunction in STZ-induced diabetic rats with hyperlipidemia^5^,^8^,^9^,^10^,^11^. However, the possible effects of DMC on hyperglycemia and the underlying mechanisms remained unexploited.

Glucagon-like peptide-1 (GLP-1), an incretin hormone derived from the transcription of proglucagon gene (GCG), is released from the intestinal endocrine L cells in response to nutritional stimuli^12^. One of the important functions of GLP-1 is to improve glucose metabolism after meals by inhibiting gastric motility and reducing appetite. As an anti-hyperglycemic hormone it acts by stimulating glucose-dependent insulin secretion^13^,^14^,^15^. In addition, GLP-1 inhibits gastric secretion and regulates glucose homeostasis in the gastrointestinal tract after meals and is required for normal glucose tolerance^16^,^17^. Glucose-dependent insulinotropic polypeptide (GIP) is a 42-amino acid peptide localized to the distal intestine, where exists a population of K-/L cells that produce both GIP and GLP-1. Recently, it is widely recognized that GIP increases insulin secretion from β-cells in a glucose-dependent manner and GIP could be manipulated for therapeutic benefit^18^,^19^. GLP-1 and GIP levels are likely to be impaired in diabetes and the regulation of the GLP-1 and GIP secretion plays crucial role and represents a promising therapeutic strategy for diabetes. Previous studies have documented the importance of hypoglycemic drugs on increasing GLP-1 and GIP release^20^,^21^,^22^. Transcription factor 7-like 2 (TCF7L2) is a risk factor of diabetes that is linked to a large variety of diseases^23^. For years, conventional wisdom held that in ileum endocrine L-cell lines, TCF7L2 controls the transcription of the proglucagon gene (GCG) that encodes GLP-1 and the GIP gene^24^,^25^. The glycogen synthasekinase-3β (GSK-3β) is involved in the regulation of glycogen synthesis and aberrant activity of GSK-3β has been implicated in diabetes^26^. The GSK-3β/β-catenin signaling pathway has a significant role on TCF7L2 transcription^27^. These published studies allow for a formulization of GSK-3β/β-catenin → TCF7L2 → GCG + GIP → blood glucose as a glucose homeostasis regulatory pathway^26^.

The present study aimed to examine this notion by investigating whether DMC possesses an anti-hyperglycemic property, in addition to its well-established anti-hyperlipidemic effect, and to decipher the underlying mechanisms of DMC in a rat model of diabetes mellitus.

## Results

### Hyperglycemic efficacies of DMC in diabetic rats

**Figure 1:**
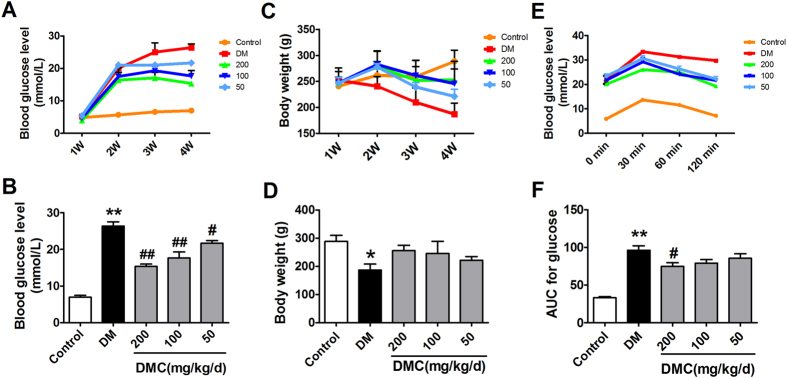
OGTT, blood glucose and body weight. (**A**) Blood glucose concentrations measured every week for 4 weeks post STZ (streptozotocin) injection to establish diabetes mellitus (DM). (**B**) Blood glucose concentrations 4 weeks post STZ injection and administration of Daming Capsule (DMC), a traditional Chinese medicine. (**C**) Body weight measured for 4 weeks after the STZ injection. (**D**) Body weight 4 weeks post STZ injection and DMC treatment. (**E**) Results of oral glucose tolerance test (OGTT) in the Control, DM and DMC groups. (**F**) Area under the curve (AUC) for OGTT. *p < 0.05, **p < 0.01 vs Control, ^#^p < 0.05, ^##^p < 0.01 vs DM; mean ± SEM.

In diabetic rats induced by STZ and fed with the high-fat diet, the fasting blood glucose (FBG) levels increased significantly and glucose tolerance declined, accompanied by a decrease in body weight and an increase in food intake, indicating the successful establishment of diabetes with typical hyperglycemia (Table 1). Administration of DMC produced strong anti-hyperglycemic effects. First, DMC effectively decreased FBG levels in DM rats, which was accompanied by an increase of body weight (Fig. 1A–D). Second, DMC also improved glucose tolerance as indicated by the reduced glucose contents within the testing period (areas under the curve; Fig. 1E,F).

### Hypolipidemic efficacies of DMC in diabetic rats

**Figure 2:**
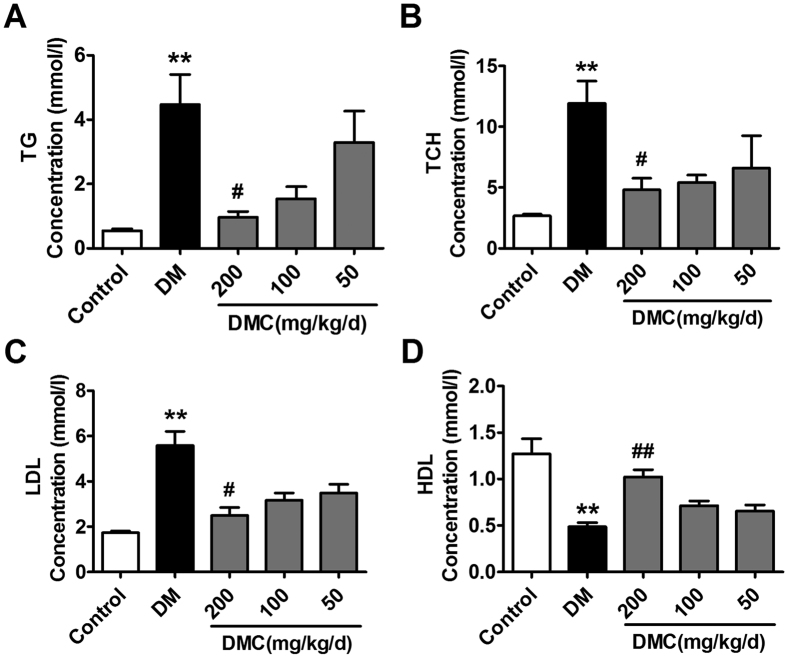
Biochemical estimations of TG, TCH, LDL and HDL. (**A–D**) TG, TCH, LDL and HDL concentrations in the serum of the Control, DM and DMC groups. **p < 0.01 vs Control, ^#^p < 0.05, ^##^p < 0.01 vs DM; mean ± SEM.

Blood triglycerides (TG), total cholesterol (TCH) and low density lipoprotein (LDL) were all significantly elevated whereas high density lipoprotein (HDL) was decreased in the DM group (Fig. 2). DMC treatment dramatically restored these adverse changes, confirming its anti-hyperlipidemic property in the setting of diabetes.

### Restoration of reduced GLP-1 and GIP levels by DMC in diabetic rats

**Figure 3:**
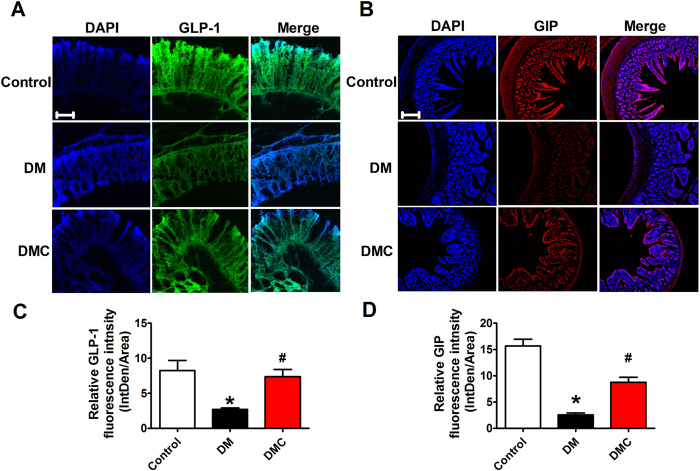
Expression of GLP-1 and GIP in ileum. (**A**) Images (×400) of immunofluorescence staining of GLP-1 (glucagon-like peptide-1) and GIP (gastric inhibitory polypeptide) expression in ileum in the Control, DM and DMC groups. (**B**) Immunofluorescence results (×400) indicating GIP expression. (scale bar: 20μm). (**C**) Quantification of the GLP-1 fluorescence intensity (integrated density per stained area). (**D**) Quantification of the GIP fluorescence intensity (integrated density per stained area). *p < 0.05 vs Control, ^#^p < 0.05 vs DM; mean ± SEM.

After establishing the hypoglycemic efficacy of DMC, we wanted to explore how this lipid-lowering drug produces its beneficial actions against diabetic hyperglycemia. It has been mentioned above that GLP-1 (an anti-hyperglycemic hormone) and GIP (a gastric inhibitory polypeptide) play important role in glucose homeostasis and many hypoglycemic drugs act by increasing GLP-1 and GIP releases^20^,^21^,^22^. We therefore sought to examine if DMC possesses similar properties. To this end, we assessed the effects of DMC on GLP-1 and GIP levels in ileum of DM rats. As shown in Fig. 3A–D with immunofluorescence staining, the abundance of GLP-1 and GIP expression in the DM group were decreased remarkably relative to healthy controls and this reduced abundance was corrected by DMC treatment.

**Figure 4:**
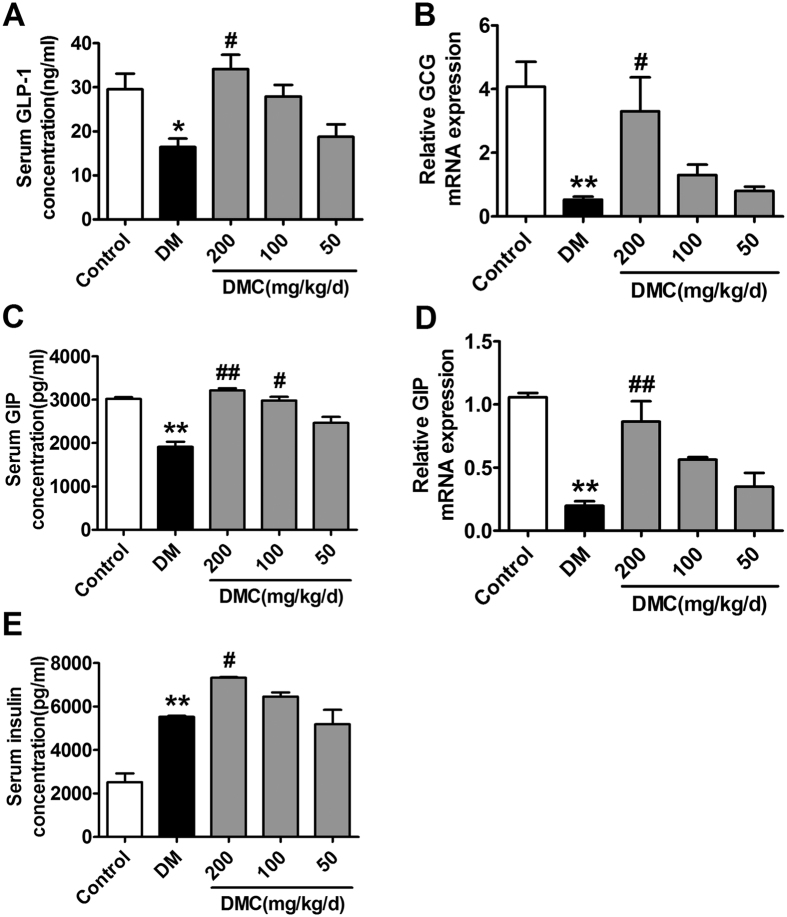
GLP-1 and GIP concentrations in the serum and mRNA levels of GCG and GIP. (**A**) GLP-1 concentrations in the serum in the in the Control, DM and DMC groups. (**B**) GCG (the gene encoding GLP-1) mRNA levels in ileum. (**C**) GIP concentrations in the serum in the five groups. (**D**) GIP mRNA levels in ileum. (**E**) Insulin levels in the serum in the five groups. *p < 0.05, **p < 0.01 vs Control, ^#^p < 0.05, ^##^p < 0.01 vs DM; mean ± SEM.

Moreover, the serum concentrations of GLP-1 and GIP were also drastically decreased by approximately 50% and 60%, respectively, as determined by enzyme-linked immunosorbent assay. Notably, DMC (200 mg/kg/d) considerably restored the diminished serum levels of GLP-1 and GIP (Fig. 4A,C). Furthermore, DMC at a dosage of 200 mg/kg/d significantly increased the mRNA levels of GCG (the GLP-1-coding gene) and GIP (Fig. 4B,D). As a result, the insulin level in the serum was higher in the DMC/DM group compared with the DM rats without DMC treatment (Fig. 4E).

### Signaling mechanisms for the anti-diabetic properties of DMC in diabetic rats

**Figure 5:**
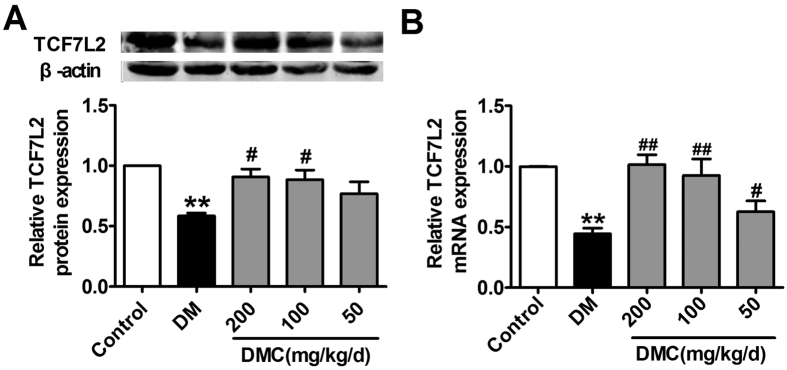
Effects of DMC on TCF7L2 expression in ileum of diabetic rats. (**A**) TCF7L2 (transcription factor 7-like 2) expression measured by the western blot in the Control, DM and DMC groups. (**B**) TCF7L2 mRNA expression detected by real-time PCR in the 5 groups. **p < 0.01 vs Control, ^#^p < 0.05, ^##^p < 0.01 vs DM; mean ± SEM.

While the above results strongly indicated the beneficial effects of DMC against hyperglycemic stress and the rescue of GLP-1 and GIP dysregulation as a possible downstream mechanism for the anti-hyperglycemic action, it remained unresolved as to how DMC acts on the expression of GCG and GIP. We chose to assess whether TCF7L2 plays a role in mediating the transcriptional activation of GCG and GIP by DMC, as this transcription factor has been proven to control the transcription of the genes encoding GLP-1 and GIP in the gut and brain^24^,^25^. The evidence we obtained was that TCF7L2 expression decreased dramatically in the DM group, and this diabetic downregulation was essentially normalized by DMC (Fig. 5A,B).

**Figure 6:**
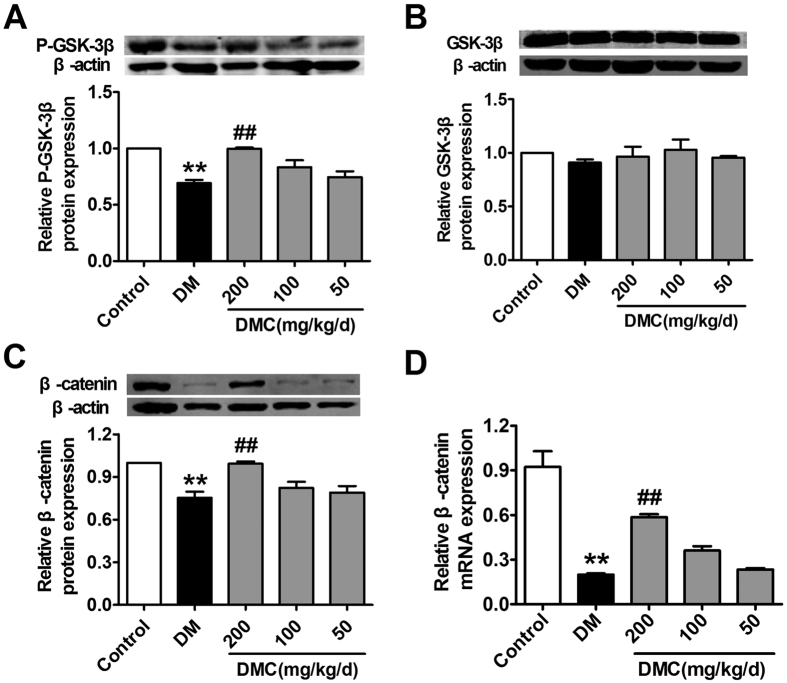
Western blot analysis of GSK-3β and β-catenin protein levels. (**A**) Relative protein levels of the phosphorylated form (or active form) GSK-3β (p-GSK-3β at Ser-9). (**B**) Total protein levels of GSK-3β. (**C**) Relative protein levels of β-catenin. (**D**) Relative mRNA levels of β-catenin in the 5 groups (n = 3). *p < 0.05, **p < 0.01 vs Control, ^#^p < 0.05, ^##^ p < 0.01 vs DM; mean ± SEM.

The next question we asked was how DMC upregulates TCF7L2 expression. It has been documented that the GSK-3β/β-catenin signaling pathway plays an important role in TCF7L2 transcriptional activation^28^,^29^. There is thus a possibility that DMC acts on TCF7L2 expression through the GSK-3β/β-catenin signaling pathway. Consistently, our western blot results revealed considerable inhibition of GSK-3β activity in DM rats as reflected by the decreased phosphorylated form of GSK-3β, and downregulation of β-catenin expression as well (Fig. 6A,B). Notably, DMC was able to rescue the reduced GSK-3β activity and restored the downregulated β-catenin expression at both mRNA and protein levels (Fig. 6C,D).

**Figure 7:**
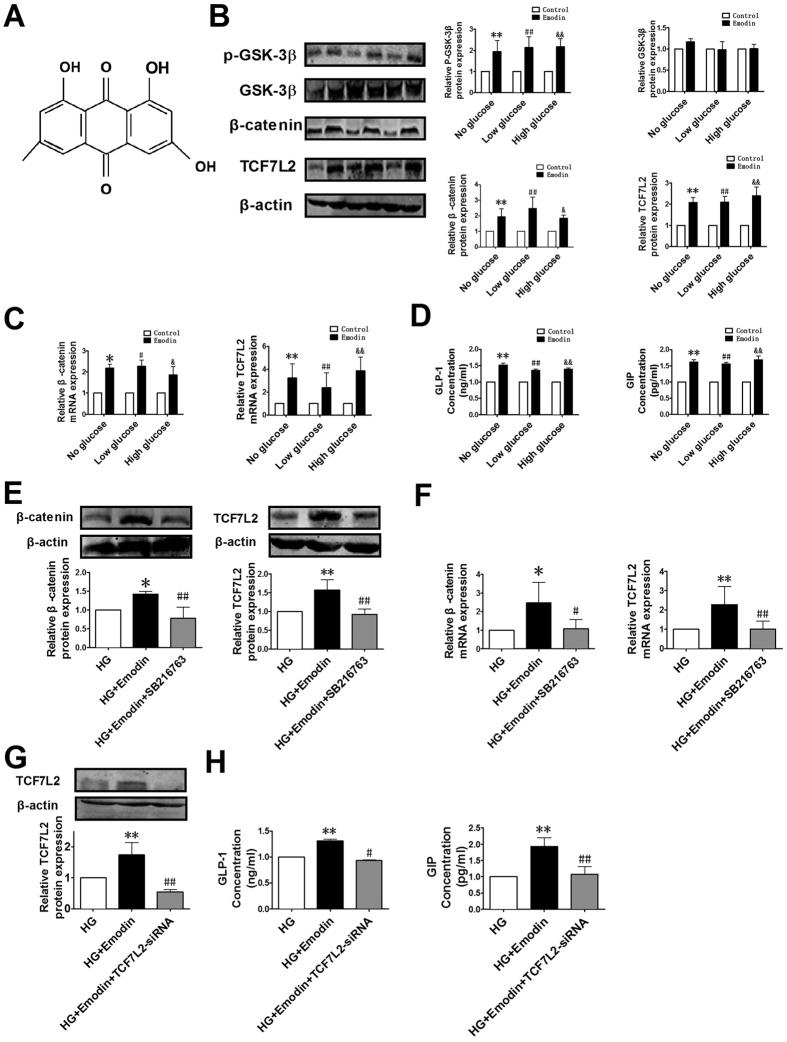
Alterations of the protein levels of the components of the GSK-3β—β-catenin—TCF7L2—GLP-1 axis under varying glucose conditions. (**A**) Molecular structure of Emodin. (**B**) Relative protein levels of p-GSK-3β, GSK-3β, β-catenin and TCF7L2 in Control and Emodin groups under different glucose conditions, detected by the western blot analysis. (**C**) Relative mRNA expression of β-catenin and TCF7L2 in Control and Emodin groups under different glucose conditions, detected by real-time PCR. (**D**) GLP-1 and GIP concentration measured by ELISA in the cell supernatant in Control and Emodin groups under different glucose conditions. *p < 0.05, **p < 0.01 vs control under no glucose, ^#^p < 0.05, ^##^p < 0.01 vs control under low glucose, &p < 0.05, && p < 0.01 vs control under high glucose; mean ± SEM. (**E,F**) Inhibition of TCF7L2 and GSK-3β abolished DMC’s effects. (**E**) Effects of emodin (the main component of DMC) and SB216763 (a specific GSK-3β inhibitor) on the protein levels of β-catenin and TCF7L2 under HG (high glucose) conditions. (**F**) Relative mRNA expression of β-catenin and TCF7L2 in the three groups. (**G**) Relative protein expression of TCF7L2 in HG, HG + emodin and HG + emodin + TCF7L2 SiRNA groups. (**H**) GLP-1 and GIP concentration measured by ELISA in the cell supernatant in the three groups. *p < 0.05, **p < 0.01 vs HG, #p < 0.05, ##p < 0.01 vs HG + Emodin; mean ± SEM.

Together the above results, it appears that DMC elicited its anti-hyperglycemic effects by enhancing GSK-3β activity and upregulating β-catenin expression, which in turn transactivated TCF7L2 that subsequently stimulated the secretion of GLP-1 and GIP leading to hypoglycemia. To further test this notion, we employed pharmacological tools and looked at the changes of these components in enteroendocrine cell line NCI-H716 cell, which is commonly used for measuring GLP-1 secretion^30^,^31^. Rheum Emodin is the main component of DMC (see Fig. 7A for the structure of emodin), and previous studies have shown that emodin acts as an anti-diabetic drug^32^ through regulating blood glucose by activating AMP-activated protein kinase^33^ and preventing high glucose-induced pathological process^34^. We therefore investigated the effects of emodin on the levels of p-GSK-3β, GSK-3β, β-catenin, TCF7L2, GLP-1 and GIP in NCI-H716 cell under different glucose conditions. As illustrated in Fig. 7B–D emodin increased the components in the GSK-3β/β-catenin/TCF7L2/GLP-1 + GIP pathway under normal glucose conditions and chronic high glucose insults. We then further examined the effects of DMC on β-catenin, TCF7L2, GLP-1 and GIP in the presence of a specific GSK-3β inhibitor SB216763 (10 μM, applied 30 min before the experimental measurements) to knockdown phosphorylation of GSK-3β. Our results showed that the ability of DMC to increase the expression of β-catenin and TCF7L2 was eliminated by inhibition of GSK-3β phosphorylation (Fig. 7E,F). Moreover, as depicted in Fig. 7G,H, silence of TCF7L2 by its siRNA greatly blunted the stimulating effect of DMC on GLP-1 and GIP release, indicating that TCF7L2 mediated GLP-1 and GIP production induced by DMC.

## Discussion

**Figure 8:**
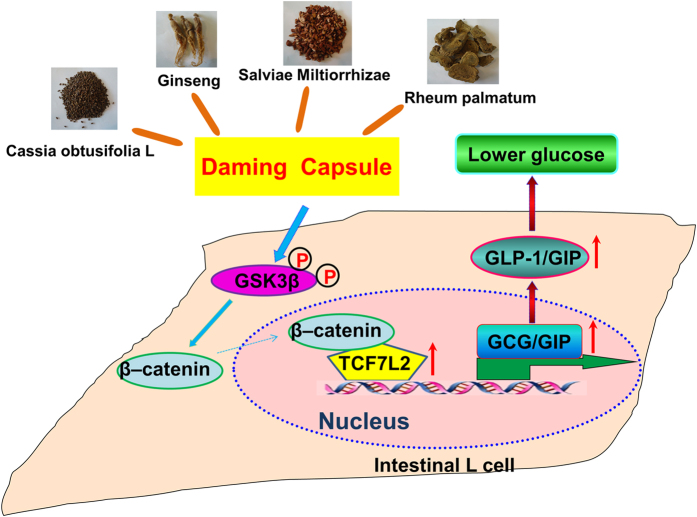
Schematic diagram showing the anti-diabetic properties of DMC. DMC is an effective strategy to lower blood glucose by upregulating the GLP-1 and GIP secretion and increase the GCG, GIP transcription by enhancing the TCF7L2 expression through the GSK-3β/β-catenin pathway.

The present study unraveled a novel pharmacological property of DMC: the anti-hyperglycemic efficacy, in addition to its long-recognized anti-hyperlipidemic effect, in a mouse model of DM. Our experiments have also presented the evidence for restoration of diabetic diminishment of GLP-1 and GIP secretion as a possible mechanism for the ability of DMC to maintain glucose homeostasis. Furthermore, our results indicated that the DMC-induced enhancement of GSK-3β activity and upregulation of β-catenin and TCF7L2 expression might account for the restoration of GLP-1 and GIP levels. On the basis of these findings, we came up with the flowchart DMC → GSK-3β/β-catenin pathway↑ → TCF7L2↑ → GCG/GIP→ GLP-1/GIP secretion↑ → blood glucose↓ as a pathway that explains for the anti-hyperglycemic property of DMC, as summarized schematically in Fig. 8.

DM is a kind of metabolic diseases in which a person has hyperglycemia and decline in insulin secretion as common manifestations, either because of decline in β-cell function, or loss of response of cells to insulin. DM can cause a large number of serious complications including diabetic neuropathy, diabetic nephropathy (chronic renal failure), cardiovascular diseases and retinal damage. TCM offers significant advantages in treating chronic diseases with little adverse side effects. Several TCMs have been documented to have beneficial effects on DM and the associated complications^35^. Huanglian Jiedu decoction and Ge-Gen-Qin-Lian decoction have been shown to produce anti-DM effects in animal experiments^36^,^37^. The outcomes from a randomized, multicentre, open-label, parallel-group trial demonstrate the efficacy and safety profile of DCM in lowering total cholesterol (TCH) and low density lipoprotein (LDL) in hypercholesterolemia patients^5^. In animals fed with a high-fat diet, DMC was found to protect the aorta from endothelial dysfunction by upregulating eNOS^11^. A study demonstrated that DMC is able to reverse the prolonged QT and PR interval and improve cardiac function in diabetic rats, and such a cardioprotective effect is attributable to its ability to modulate the expression of a potassium channel Kv4.2^9^. However, whether DMC possesses glucose-regulatory efficacy has remained unstudied prior to the present study. Our data suggested that DMC can elicit dual actions in the setting of DM: anti-hyperglycemic and anti-hyperlipidemic efficacies that together are expected to afford synergistic anti-diabetic effects. While the anti-hyperlipidemic action of DMC is the primary property of TCM this as it is designed for this purpose, the present study is the first to show the glucose-regulatory role of DMC.

The essential roles of GLP-1 and GIP in maintaining blood glucose homeostasis have been widely recognized. GLP-1 is an incretin secreted from the intestinal L cells, which regulates blood glucose homeostasis via stimulation of insulin secretion, attenuation of gastric emptying and promotion of satiety^17^,^38^,^39^. Many drugs and Chinese herbal medicines improve diabetes by regulating GLP-1^40^. The recently emerged agents like dipeptidyl peptidase-4 (DPP-4) inhibitors and the GLP-1 analogues have been shown to be effective in treating DM^41^. A recent study demonstrated that green tea extract derived from the plant Camellia significantly improves insulin resistance by increasing GLP-1^42^. Huang Lian Jie Du Decoction exerts its anti-diabetic effects by modulating GLP-1 in rats^36^. GLP-1 is encoded by the GCG gene which is expressed in pancreatic cells and ileum endocrine L cells. It should be pointed out that although GLP-1 stimulates insulin secretion, whether serum GLP-1 level is declined in diabetic patients remains controversial. GIP stimulates insulin secretion from the pancreatic islets^43^. We found here that serum GLP-1 and GIP in DM rats were significantly declined compared with that in the control littermates, and DMC treatment was able to restore the serum level of GLP-1 and GIP levels. The increased levels are consistent with higher expression of GCG and GIP in the DMC treatment group compared with the DM group. Our findings provided insight into a molecular and signaling mechanism for the efficacy of DMC in clearing up the excessive blood sugar.

TCF7L2 is known to promote GCG and GIP transcription in ileum and insulin secretion as well^25^,^44^. Our data indeed provided evidence in support of this notion; we found that DMC normalized the decreased level of TCF7L2 through the GSK-3β/β-catenin pathway and enhanced GCG and GIP transcription in a DM animal model. In addition, our *in vitro* experiments found that after inhibition of the phosphorylation of GSK-3β, DMC failed to restore β-catenin and TCF7L2 levels. Moreover, inhibition of the TCF7L2 can abolish the effect of DMC on GLP-1 and GIP release in NCI-H716 cell line under high glucose conditions. These data all support that DMC directly targets GSK-3β/β-catenin/TCF7L2/GLP-1 axis and lower the blood glucose.

The salient findings in the present study supported a novel pharmacological effect of DMC: effectively lowering the blood glucose in STZ-induced diabetic rats. Moreover, our findings unraveled the enhancement of GLP-1 and GIP secretion into blood stream as a biochemical mechanism for the anti-diabetic property of DMC. Furthermore, our results revealed the ability of DMC to upregulate the GSK-3β/β-catenin pathway and TCF7L2 so as to enhance GLP-1 and GIP levels thereby blood glucose reduction. We therefore proposed DMC →GSK-3β/β-catenin pathway↑ → TCF7L2↑ → GCG, GIP→ GLP-1, GIP secretion↑ → blood glucose↓ as a regulatory pathway of blood glucose homeostasis, which underlies the anti-hyperglycemic property of DMC. However, we are well aware that our results do not allow us to deduce a definite mechanism for anti-hyperglycemic property of DMC. Alternative signal pathways such as AMPK, STAT3 signaling could be involved in the regulation of the blood glucose homeostasis by DMC, which merits future investigations.

## Methods

### Ethics statement

This study was specifically approved by the ethic committees of Harbin Medical University. The experimental procedures used in our study were approved by the Animal Care and Use Committee of Harbin Medical University (HMUIRB-2008-06), and confirmed with the Guide for the Care and Use of Laboratory Animals published by the US National Institutes of Health (NIH Publication No. 85-23, revised 1996).

### Plant material and preparation

DMC (Lot no. 20120301) was used in the study and the formulation of DMC was designed by Professor Baofeng Yang in the Department of Pharmacology of the Harbin Medical University (Patent No. ZL03109063.X). Rumex gmelinii Turcz. ex Ledeb.(Solanaceae) was harvested from Gansu Province with 1 year cultivation; Salvia miltiorrhiza (Labiatae) was from Jilin Province aged 8 years; Cassia obtusifolia L.(pulse family) and Panax ginseng C. A. Meyer (Acanthopanax gracilistylus) were from Sichuan Province after 3 years of cultivation. These plant materials were all collected from September to November. The voucher specimens are: Rheum palmatum L. (1.008.1) Panax ginseng C.A. Mey (1.058.1) Salvia miltiorrhiza Bunge (1.080.1) Cassia obtusifolia L. (6.048.1), and were deposited at the Heilongjiang Food and Drug Administration (Harbin, China)^9^. DMC was produced by Harbin Yida Ltd as described previously and the preparation is described as follows briefly. The samples were dried, crushed into a fine powder and cased in capsule in a Rumex gmelinii Turcz. ex Ledeb.: Cassia obtusifolia L.: Salvia miltiorrhiza: Panax ginseng C. A. Meye ratio of 12:12:6:1^5^. All processes were superintended according to the policy of the State Food and Drug Administration of P. R. China. HPLC analysis of the total anthraquinones for quality control/quality assurance in DMC was also presented in our previous study^9^.

### Chemical compounds of Daming Capsule

Emodin (Pubchem CID: 3220); Chrysophanol (Pubchem CID: 10208); Anthraquinone (Pubchem CID: 6780); Ginsenoside Rb1 (Pubchem CID: 9898279); Tanshinone I (Pubchem CID: 114917); Nobiletin (Pubchem CID: 72344); Hesperidin (Pubchem CID: 10621); Cassiaside (Pubchem CID: 164146).

### Animals

Male Wistar rats (180–220 g body weight) were obtained from the Animal Center of the 2nd Affiliated Hospital of Harbin Medical University. Rats were kept in an animal house with controlled temperature of 23 ± 1 °C and humidity of 55 ± 5%. Animals were raised on 12 h dark-light artificial cycle under standard conditions with food and water available ad labium^45^.

### Establishment of diabetic models

Wistar rats were gavaged with high fat diet (2 ml/d) which was prepared with lard (20%), cholesterol (5%), sucrose (5%), glucose (5%) and salt (6%) and emulsified in 20% Tween 80 and 30% propylene glycol with distilled water. Diabetic rats were induced by intraperitoneal injection of 35 mg/kg/d streptozocin (STZ) from Sigma (St Louis, MO, USA) in 0.1 M citrate buffer solution (pH = 4.3) for three consecutive days^46^. Fasting blood glucose (FBG) levels were measured 72 h after STZ injection by Grace Glucometer (Grace Medical, Inc. America) and diabetic model was considered successfully established in rats with blood glucose levels higher than 16.7 mmol/l. Rats with STZ injection were randomly divided into Control group (Control), Diabetes mellitus group (DM) and DM + DMC (50, 100 and 200 mg/kg/d) groups. DMC groups were intragastrically administered with DMC for 4 weeks. The Control group was treated with an equal volume of 0.9% NaCl.

### Biochemical estimations

Blood samples obtained from tail vein were used for examining FBG levels after gastric emptying for 12 h by Grace Glucometer. Blood serum was collected from artery and used for the measurements of triglycerides (TG), total cholesterol (TCH), low density lipoprotein (LDL) and high density lipoprotein (HDL) with respective assay kits (Nanjing Jiancheng Bioengineering Institute, Beijing Beihuakangtai Clinical Reagent Ltd. and Zhejiang Dongou Diagnostic Products Co., China)^47^.

### Oral glucose tolerance test (OGTT)

After 12h fasting, OGTT was performed using Grace Glucometer (Hoffmann-La Roche Ltd). Glucose was intragastrically administered (2 g/kg body weight) and blood glucose was measured at time points of 0, 30, 60 and 120 min post gavage. Glucose contents were calculated as the areas under the curve (AUC)^48^.

### Immunofluorescence staining

The ileum tissues were fixed with 4% buffered paraformaldehyde and were incubated with GLP-1 and GIP antibody (Wuhan Boster Biological Technology., LTD, China) at 4 °C overnight, followed by incubation with the secondary antibody anti-rabbit IgG (Alexa Fluor 488: Molecular Probes, Eugene, OR). The sections were stained using 40,6-diamidino-2-phenylindole (DAPI) (Beyotime, China) for 15 min at room temperature^49^. Immunofluorescence was examined under a fluorescence microscope (Nikon 80i, Japan).

### Enzyme-linked immunosorbent assay measurement

Serum contents of GLP-1 (Millipore, EGLP-35K), GIP (rats: uscn-CEA882Ra, human: Elabscience: E-EL-H0148c) and insulin (uscn-CEA448Ra) were detected using ELISA kit according to the manufacturer’s instructions^50^.

### RNA isolation and real-time PCR

The animals were euthanized and the ileum was immediately dissected, frozen in liquid nitrogen and stored at –80 °C. Ileum tissues were carefully transferred into an RNase-free tube. Total RNA was isolated from the preparations in 1 ml of TRIzol reagent (invitrogen, USA). cDNA synthesis was performed with the High Capacity cDNA Reverse Transcription Kit (Applied Biosystems, Cat. #4368814) according to the manufacturer’s instructions. The SYBR Green PCR Master Mix Kit (Applied Biosystems, Cat. #4309155) was used for relative quantification of RNAs. GAPDH was used as an internal control. Real-time PCR was performed on 7500 FAST Real-Time PCR System (Applied Biosystems, Carlsbad, CA) for 40 cycles. The primers used in the study are provided in Supplemental Table 2^51^,^52^.

### Western blot analysis

Tissues dissected from the ileum of the STZ-induced rats were lysed in 300 μl buffer containing 1% protease inhibitor solution, and then centrifuged to collect the soluble proteins. The protein content was determined using the BCA Protein Assay kit by spectrophotometer (Bio-Rad, Mississauga, ON, Canada). Protein samples (60 μg) were resolved on 10% SDS-PAGE gels and transferred to nitrocellulose membranes^53^. After blocking in 5% nonfat milk for 2 h at room temperature, membranes were incubated with the indicated primary antibodies against GSK-3β (Cat No. WL01593, Wanleibio, Shenyang, P.R. China), p-GSK-3β (Ser-9) (Cat No. ap0039, ABclonal Biotech Co., Ltd, Wuhan, Hubei, P.R. China), β -catenin (Cat No. 610154, BD Biosciences, San Jose, CA, USA), and TCF7L2 (Cat No. Ab76151, Abcam, Cambridge, MA, USA) diluted at 1:500 in PBS buffer 4 °C overnight. Membranes were then washed in phosphate-buffered saline with Tween 20 and incubated for 1h with the fluorescence-conjugated anti-rabbit IgG secondary antibody (1:10000). Western blot bands were quantified using Odyssey v1.2 software by measuring the band intensity (Area × OD) for each group and normalized to β-actin (Santa Cruz)^54^. The normalized expression levels of target proteins are presented as fold changes normalized to the control values^51^.

### Cell culture and transfection

NCI-h716 cells were seeded in RPMI 1640 (Hyclone, Logan, UT, USA) containing 10% fetal bovine serum (Hyclone, Logan, UT, USA) and used for all experiments. Cells were incubated in humidified air with 5% CO_2_ at 37 °C. Cells were transfected with Opti-MEM medium (Invitrogen CA, USA) and lipo2000 (Invitrogen CA, USA). Cells were divided into three groups: 25 mM High glucose (HG) group, HG + Emodin (100 μM) (Cat No. JOT-10023, Chengdu Pufei De Biotech Co., Ltd) group, and HG + Emodin + siRNA/inhibitor group. For the experiments involving silence of TCF7L2, cells were transfected with TCF7L2-targeting siRNA oligonucleotide synthesized by Shanghai GenePharma Co., Ltd. For the experiments involving SB216763 (a specific GSK3β inhibitor; ab120202; Abcam, Cambridge, MA, USA), cells were incubated with the agent at 37 °C for 24 h.

### Data analysis

Data are presented as mean ± SEM. One-way analysis of variance (ANOVA) test was performed for multiple comparisons by GraphPad Prism 5.0. Quantification of integrated density per stained area was performed using image J. P-values less than 0.05 were considered statistically significant.

## Additional Information

**How to cite this article**: Zhang, Y. *et al*. The anti-hyperglycemic efficacy of a lipid-lowering drug Daming capsule and the underlying signaling mechanisms in a rat model of diabetes mellitus. *Sci. Rep*. **6**, 34284; doi: 10.1038/srep34284 (2016).

## Supplementary Material

Supplementary Information

## Figures and Tables

**Table 1:** Body weight, blood glucose, water intake and food intake in Control and DM group.

Parameter	Control (n = 8)	DM group (n = 8)
Body weight before	240.7 ± 6.5	236.1 ± 2.6
Body weight	288.2 ± 8.6	226.1 ± 3.2^**^
Glucose before	4.9 ± 0.1	4.8 ± 0.05
Glucose	7.0 ± 0.2	16.2 ± 0.3^**^
Water intake before	62.9 ± 0.1	61.0 ± 1.3
Water intake after	68.2 ± 1.1	122.0 ± 0.4^**^
Food intake before	20.2 ± 0.3	25.8 ± 1.8
Food intake after	20.9 ± 0.1	22.3 ± 0.2

Parameters in the Control and DM group indicating the successfully established diabetic models. **p < 0.01 vs Control.
