# Supplementary material for: The anti-hyperglycemic efficacy of a lipid-lowering drug Daming capsule and the underlying signaling mechanisms in a rat model of diabetes mellitus
Source: Sci Rep. 2016 Oct 10;6:34284. doi: 10.1038/srep34284 (PMC5056381; doi:10.1038/srep34284)
Supplement: Supplementary Information [file srep34284-s1.doc]

**The anti-hyperglycemic efficacy of a lipid-lowering drug Daming capsule** **and the underlying signaling mechanisms in a rat model of diabetes mellitus**

Yong Zhang^1, 2, 3*^, Xiaoguang Li^1,4*^, Jiamin Li^1^, Qingwei Zhang^1^, Xiaohui Chen^1^, Xin Liu^1^, Yue Zhang^1^, Haiying Zhang^1^, Huan Yang^1^, Yingying Hu^1^, Xianxian Wu^1^, Xin Li^1^, Jiaming Ju^1^ & Baofeng Yang^1, 2, 5^

*^1^Department of Pharmacology (State-Province Key Laboratories of Biomedicine-Pharmaceutics of China, Key Laboratory of Cardiovascular Medicine Research, Ministry of Education), College of Pharmacy, Harbin Medical University, Harbin, 150081, China*

*^2^Institute of Cardiovascular Research, Harbin Medical University, Harbin 150081, China*

*^3^Institute of Metabolic Disease, Heilongjiang Academy of Medical Science, Harbin 150086, China*

*^4^Current address: Department of biological chemistry, Johns Hopkins School of Medicine, Baltimore, Maryland, United States of America.*

*^5^Department of Pharmacology and Therapeutics, Melbourne School of Biomedical Sciences, Faculty of Medicine, Dentistry and Health Sciences, University of Melbourne, Melbourne, Australia.*

*These authors contributed equally to this work. Correspondence and requests for materials should be addressed to Yong Zhang (email: hmuzhangyong@hotmail.com) or Baofeng Yang (email: yangbf@ems.hrbmu.edu.cn).

**Supplemental table 1. The Chinese medicines contained in DMC.**

| Latin name (Chinese name) | The part used | Collected place | Voucher numbers |
| --- | --- | --- | --- |
| Rheum palmatum L. (Da Huang) | Root | Gansu | 10081 |
| Cassia obtusifolia L. (Jue Ming Zi) | Fruit | Sichuan | 60481 |
| Salvia miltiorrhiza Bunge. (Dan Shen) | Root | Jilin | 10801 |
| Panax ginseng C. A. Mey. (Ren Shen) | Root | Sichuan | 10581 |

**Supplemental table 2. Nucleotide sequence of primers for real-t**ime PCR.

| Gene | Primer Sequence | GenBank No. |
| --- | --- | --- |
| Rat-GIP | Forward: AATGCCAAAACACCTTGGAG | NM_019630.3 |
|  | Reversed: TCTCTCCAAGATCCCCATTG |  |
| Rat-β-Catenin | Forward: CCGAGGACTCAATACCATTC | NM_053357.2 |
|  | Reversed: CAGACATTCGGAATAGAACAG |  |
| Human-β-Catenin | Forward: GTGCTGAAGGTGCTATCTGT  Reversed: ACCTTCCATCCCTTCCTGTTT | NM_001098209.1 |
| Rat-GCG | Forward: AGGCAATGTTGTTCCGGTTCCT | NM_012707.2 |
|  | Reversed:  AACGCCAGATCATTCCCA |  |
| Rat-TCF7L2 | Forward: CAGCAAGGTCAGCCTGTGTA  Reversed: CACCACCTTCGCTCTCATCT | NM_001191052.1 |
| Human-TCF7L2 | Forward: TCCCACCACATCATACGCTA  Reversed: CTACGACCTTTGCTCTCATTTC | NM_001146274.1 |
| GAPDH | Forward: AAGAAGGTGGTGAAGCAGGC | NM_017008.4 |
|  | Reversed: TCCACCACCCAGTTGCTGTA |  |
